# Emerging Roles of Heat-Induced circRNAs Related to Lactogenesis in Lactating Sows

**DOI:** 10.3389/fgene.2019.01347

**Published:** 2020-02-11

**Authors:** Jiajie Sun, Haojie Zhang, Baoyu Hu, Yueqin Xie, Dongyang Wang, Jinzhi Zhang, Ting Chen, Junyi Luo, Songbo Wang, Qinyan Jiang, Qianyun Xi, Zujing Chen, Yongliang Zhang

**Affiliations:** ^1^College of Animal Science, Guangdong Provincial Key Laboratory of Animal Nutrition Control, Guangdong Engineering & Research Center for Woody Fodder Plants, National Engineering Research Center for Breeding Swine Industry, South China Agricultural University, Guangzhou, China; ^2^College of Animal Science, Zhejiang University, Hangzhou, China

**Keywords:** heat stress, lactating sow, circRNA, ceRNA, casein

## Abstract

Heat stress negatively influences milk production and disrupts normal physiological activity of lactating sows, but the precious mechanisms by which hyperthermia adversely affects milk synthesis in sows still remain for further study. Circular RNAs are a novel class of non-coding RNAs with regulatory functions in various physiological and pathological processes. The expression profiles and functions of circRNAs of sows in lactogenesis remain largely unknown. In the present study, long-term heat stress (HS) resulted in a greater concentration of serum HSP70, LDH, and IgG, as well as decreased levels of COR, SOD, and PRL. HS reduced the total solids, fat, and lactose of sow milk, and HS significantly depressed CSNαs1, CSNαs2, and CSNκ biosynthesis. Transcriptome sequencing of lactating porcine mammary glands identified 42 upregulated and 25 downregulated transcripts in HS vs. control. Functional annotation of these differentially-expressed transcripts revealed four heat-induced genes involved in lactation. Moreover, 29 upregulated and 21 downregulated circRNA candidates were found in response to HS. Forty-two positively correlated circRNA-mRNA expression patterns were constructed between the four lactogenic genes and differentially expressed circRNAs. Five circRNA-miRNA-mRNA post-transcriptional networks were identified involving genes in the HS response of lactating sows. In this study we establish a valuable resource for circRNA biology in sow lactation. Analysis of a circRNA-miRNA-mRNA network further uncovered a novel layer of post-transcriptional regulation that could be used to improve sow milk production.

## Introduction

In seasonal climates, high ambient temperature is the primary environmental stress impacting domestic animal performance, including growth, reproduction, and lactation (Das et al., 2016). In general, high-yielding animals are especially susceptible to thermal stress since they generate considerably more metabolic heat (Kadzere et al., 2002). In response to heat stress (HS), dairy animals experience a sustained reduction in appetite and nutrient uptake (Bohmanova et al., 2007) and a subsequent reallocation of energy for heat acclimation (Renaudeau et al., 2012), thereby resulting in decreased milk yield and milk quality, which negatively affects the efficiency and profitability of animal farms worldwide (Hill and Wall, 2015).

In modern swine husbandry, lactating sows have been heavily selected for increased productivity (fertility, disease resistance, feed conversion efficiency, and so on) during the last two decades, and are especially at risk of HS (Renaudeau, 2005), as they have a thermal neutral zone between 16 and 22°C (Messias de Bragança et al., 1998). It is noteworthy that high temperatures above 25° are sustained for half a year in the south of China; thus, local sows are often exposed to hot conditions. Under thermal stress, individuals normally increase respiration rates and reduce feed intake (Quiniou and Noblet, 1999), in an effort to generate a negative energy balance to promote metabolic heat loss to counter HS (Renaudeau et al., 2001; Renaudeau et al., 2012). In addition, HS also influences milk production in lactating sows, perhaps through an indirect effect associated with reduction in feed intake (Ribeiro et al., 2018); however, previous reports of Messias de Bragança et al. (1998) and Silanikove et al. (2009) suggested that there may be a direct effect of ambient temperature on mammary gland metabolism in connection with low milk yield. Thus, identifying key differences in the mammary gland of lactating sows in response to high ambient temperatures has the potential to improve the productivity of sows in adverse environments (Collier et al., 2006). The ability to use powerful genomic tools to evaluate genetic differences associated with thermal tolerance can provide important information on the underlying mechanisms of HS on lactation, and will permit the selection of sows for resistance to HS.

Circular RNAs (circRNAs) are a recently identified genetic element that are abundantly expressed, highly conserved between different animal species (Hanan et al., 2017), and are involved in the foundation of mammary gland growth and development (Zhang et al., 2015; Zhu et al., 2016), milk synthesis (Zhang C. et al., 2016), and secretion and transportation (Wang et al., 2019). HS greatly impacts circRNA biogenesis, and heat-induced circRNAs perform substantial regulatory functions through circRNA-mediated competing endogenous RNA (ceRNA) networks (Pan et al., 2018). Although patterns of circRNA expression and function have been revealed among various developmental stages and physiological conditions (Lai et al., 2018; Patop and Kadener, 2018), little is known about how HS affects circRNAs in lactation. In this study, we focused on circRNAs involved in the HS response of lactating sows, and we explored potential mechanisms underlying circRNA regulation in mammary tissue.

## Materials and Methods

### Study Design and Sample Collection

A total of 60 healthy purebred Landrace sows (2–3 parity and without genetic relationships) were separated into two balanced cohorts of 30 animals each, and HS tests were conducted at a local thoroughbred farm during December 2016 and during August 2017 (WENS Shuitai Breeding Pig Farm, Guangdong, China). All sows were fed the same commercial formula diet and raised under the same management conditions. In the experimental stage, the ambient temperatures and relative humidity were measured at 14:00pm in everyday. In details, one cohort of 30 sows was selected in the winter months with a moderate average temperature, designated as the non-heat stress (NS) population; other cohort of 30 animals was selected in the summer months with a higher average temperature, designated as the HS population. Within each cohort, the number of piglets born alive was recorded, and litter birth weights of piglets were obtained within 24 hours after farrowing. Piglets were not offered creep feed, and sow milk was the only feed available to the piglets during lactation. On day 21, weaning survival and the weights of living piglets per litter were recorded and used to calculate average daily weight gain. Blood samples (10 ml) were collected at 10:00am from fasted sows using jugular venipuncture at weaning, and ELISA kits (Nanjing Jiancheng Bioengineering Institute, Nanjing, China) were used to determine serum LDH, IgG, SOD, HSP70, COR, and PRL concentrations. Sow milk samples (approximate 20 ml) were obtained on d 3, 15, and 20 of lactation from the last two pairs of sow nipples at 10:30–11:30am in each animal, and oxytocin was used to stimulate let-down. Three milk samples from each animal was pooled equally to evaluate the effect of environmental temperature on milk composition. In each environmental group, six animals that balanced for weaned backfat thickness and weight were chosen and humanely slaughtered at 21 day postpartum, and suckled mammary glands were split down the mid-line and tissues were excised from the center portion of four glands from the fourth and fifth pairs of nipples. Connective tissue and fat were removed. Mammary tissues were cut into small pieces and snap-frozen in liquid nitrogen prior to subsequent processing. In general, the collected mammary tissues contain primarily secretory epithelial cells, with a small amount of myoepithelial cells, endothelial cells, adipocytes, fibroblasts, and immune cells (Kensinger et al., 1986). All procedures were conducted under protocols approved by the Institutional Animal Care and Use Committee of South China Agricultural University, China.

### RNA Preparation and Sequencing

Total RNA was extracted from mammary tissue and purified using Trizol reagent (Invitrogen, Carlsbad, CA), according to the manufacturer's instructions. Each RNA sample was treated with DNase I (Takara, Dalian, China) for 15 minutes at 37°C to remove residual genomic DNA. RNA quantity and purity were analyzed using a Bioanalyzer 2100 (Agilent, Palo Alto, CA), and RNA samples with Integrity Number (RIN) value ≥ 7.5 were used for further analysis. In each experimental condition, we randomly selected two RNA samples and pooled 5 μg of RNA from each sample. In total, six RNA pools were depleted of ribosomal RNA using an Ribo-Zero™ rRNA Removal Kit (Illumina, San Diego, USA), and the left poly-A^−/+^ RNA fractions were then reverse-transcribed to create the final cDNA using a mRNA-Seq sample preparation kit (Illumina, San Diego, CA). Finally, we performed paired-end sequencing on an Illumina Hiseq 4000 (LC Bio, Hangzhou, China) to yield 2 × 150 nucleotide reads, following the manufacturer's recommended protocol.

### Bioinformatics Analysis

Raw sequences quality was verified using FastQC (http://www.bioinformatics.babraham.ac.uk/projects/fastqc/), and the reads that contained adaptor contamination, low quality, and undetermined bases were removed by Cutadapt (Martin, 2011). Filtered data from each library was aligned to the *Sscrofa11.1* reference genome downloaded from Ensembl genome website (ftp://ftp.ensembl.org/pub/release-94/fasta/sus_scrofa/dna/) with TopHat v2.1.1 (Kim et al., 2013), and transcript assembly and abundance estimation were performed using Cufflinks v2.2.1 (Trapnell et al., 2012). Each assembly was then merged using Cuffmerge to create a single transcriptome annotation with known porcine genes in gtf format (ftp://ftp.ensembl.org/pub/release-94/gtf/sus_scrofa) for subsequent protein-coding gene analysis. To predict circRNA candidates, five different algorithms including CIRCexplorer2 (Zhang X. et al., 2016), circRNA_Finder (Fu and Liu, 2014), CIRI (Gao et al., 2015), find_circ (Memczak et al., 2013), and MapSplice (Wang et al., 2010) were performed on each RNAseq library. Only circRNA candidates identified by all five approaches were considered for further evaluation. Following the above primary analysis, expression levels of all coding genes in each library were estimated from the TopHat alignments as fragments per kilobase of exon per million mapped reads (FPKM), and Cuffdiff, included in the Cufflinks package, was used to compare expression levels between NS and HS with a false discovery rate (FDR) value < 0.05. The abundance of circRNA candidates was calculated with back-spliced junction read count (Zhang et al., 2014). Then, the edgeR software package (Robinson et al., 2010) was used to examine the differential expression (DE) of circRNA candidates with *P* value < 0.05 and fold change ≥ 2. Finally, Biological Processes GO terms and KEGG pathway analysis of the DE genes were performed using DAVID gene functional classification (https://david.ncifcrf.gov/).

### CeRNA Network Construction

Putative interactions between the DE circRNAs and lactation-related coding genes that responded to HS in our paper were evaluated by competing to bind with shared miRNAs. Porcine mature miRNAs published in miRBase (http://www.mirbase.org/) were prepared for further analysis. In details, the construction of ceRNA networks included three steps: (1) the correlations between DE circRNAs and lactation-related genes were calculated by the Pearson test, and only nodes in positive circRNA-gene interactions were retained; (2) the circRNA-miRNA and mRNA-miRNA interactions were predicted by miRanda algorithm (Betel et al., 2010) with with energies of ≤ −20.0 kcal/mol and no mismatch in the seed region (positions 2–8 in the 5′ end); (3) potential circRNA-miRNA-gene interactions were established and visualized using Cytoscape V3.4 (http://cytoscape.org/).

### Validation of Sequencing Data by Reverse transcription quantitative real-time polymerase chain reaction (RT-qPCR)

Total RNA from the NS and HS samples were isolated with Trizol reagent (Invitrogen, Carlsbad, CA), and cDNA synthesis was conducted using PrimeScript RT reagent Kit (Takara, Dalian, China) with random hexamers. Quantitative PCR was used to analyze the expression changes of the chosen transcripts with SYBR Premix Ex Taq II (Takara). All primers are listed in **Table S1**, and final expression data were calculated using the 2^−ΔCt^ method using porcine GAPDH as a reference gene.

### Sequencing Data Submission

All sequencing raw datasets have been deposited into the National Center for Biotechnology Information (NCBI) Sequence Read Archive (SRA) database (https://www.ncbi.nlm.nih.gov/sra/) with the BioProject accession number PRJNA578241.

### Statistical Analysis

The statistical analysis was performed using by SPSS 17.0 Statistics Software (Chicago, IL, USA). The results of ELISA and RT-qPCR analysis between two groups were compared with independent t-test; the correlation analyses of DE circRNAs with lactation-related coding genes were tested by function cor (x, y, use = “p”), and illustrated with function labeledHeatmap (Matrix, xLabels, yLabels) in R package WGCNA (http://127.0.0.1:11153/library/stats/html/cor.html).

## Results

### Sow and Litter Performance

**Figure 1:**
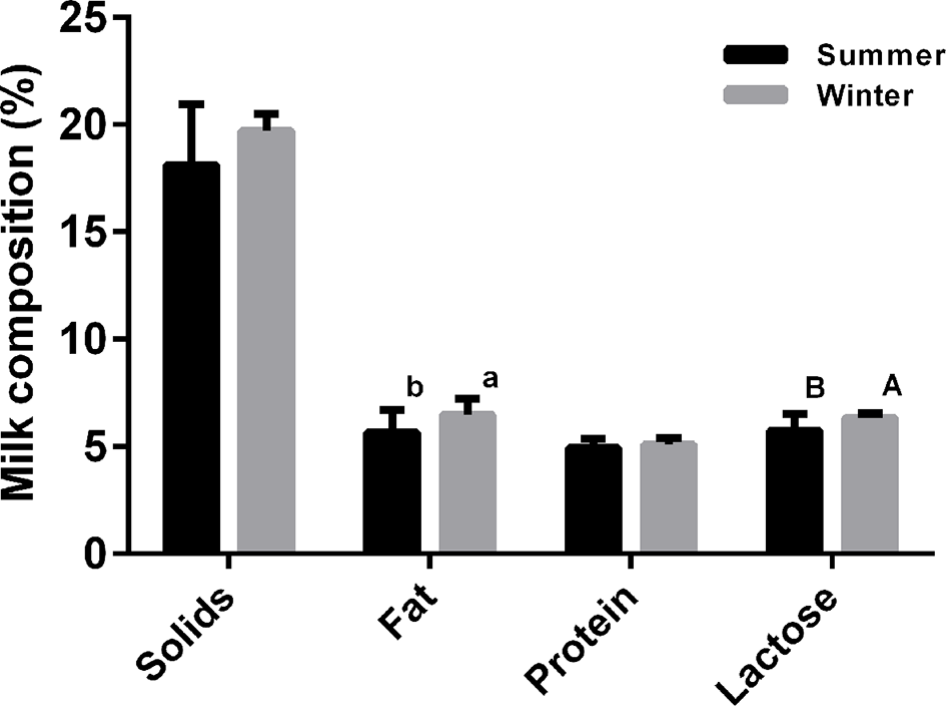
Effect of thermal stress on milk composition of lactating sows. a and b denote values that differ significantly at *P* < 0.05, and A and B denote values that differ significantly at *P* < 0.01 (N = 30).

This study was performed during December 2016 and during August 2017. During the winter experimental stage, ambient temperatures and relative humidity averaged 14.3 ± 0.81°C and 65.0% ± 0.69%, while the corresponding values for the hot season were 32.7 ± 1.40°C and 76.1% ± 0.38%, respectively. The effects of ambient temperature on the serum stress-associated variables of lactating sows are presented in **Figure S1**. Blood heat shock protein 70 (HSP70), lactate dehydrogenase (LDH), and immunoglobulin G (IgG) levels were significantly higher in the HS cohort compared with the NS group (*P* < 0.05). In contrast, serum superoxide dismutase (SOD) and prolactin (PRL) concentrations were lower in the HS population (*P* < 0.05). The high temperature group had significantly lower serum cortisol (COR) concentration than the NS group (*P* < 0.05). In addition, there was no effect of thermal stress on the number of piglets born alive, litter birth weights, or piglets alive per litter at weaning (*P* > 0.05, **Table 1**). In contrast, litter weights at weaning were significantly higher in the NS cohort than the HS cohort (*P* < 0.05). And a reduced average daily weight gain of piglets (193.9 ± 2.19 vs. 218.0 ± 1.89 g, respectively, for the HS and NS cohorts) was associated with the environment with a high temperature. Thermal stress also altered the milk composition of lactating sows. In particular, sow milk had less butterfat, and lactose when sows lactated in the hot environment (*P* < 0.05), and milk in the HS group had a downward trend for total milk solids and milk protein levels (**Figure 1**). HS individuals tended to have much lower casein alpha s1 (CSNαs1) and s2 (CSNαs2) distributions (*P* < 0.01), while casein beta (CSNβ) and casein kappa (CSNκ) concentrations decreased from 343.59 ± 6.42 μg/ml and 7.47 ± 0.16 μg/ml in the HS group to 259.14 ± 7.96 μg/ml and 6.35 ± 0.12 μg/ml in the NS group (*P* < 0.01), respectively. Under HS, we found no overall differences in whey acidic protein (WAP) concentrations between the NS and HS groups (*P* = 0.067), although there was a slightly decreasing trend in the HS group (**Table 2**).

**Table 1:** Production traits of tested sows and piglets between summer and winter.

Variables	Summer (N = 30)	Winter (N = 30)
Number born alive	10.8 ± 0.13	10.9 ± 0.09
Weaning survival	10.4 ± 0.13	10.5 ± 0.11
Litter birth weight, kg	14.9 ± 0.18	15.2 ± 0.17
Weight of weaning litter, kg	61.3 ± 0.73^B^	67.2 ± 1.59^A^
Average daily gain, g	193.9 ± 2.19^B^	218.0 ± 1.89^A^

A and B denote values that differ significantly at P < 0.01.

**Table 2:** Effect of thermal stress on lactoprotein distribution of lactating sows.

Variables	Summer (N = 30)	Winter (N = 30)
CSNαs1, μg/ml	592.03 ± 23.31^B^	693.63 ± 16.35^A^
CSNαs2, μg/ml	282.31 ± 15.00^B^	409.85 ± 9.27^A^
CSNβ, μg/ml	259.14 ± 7.96^B^	343.59 ± 6.42^A^
CSNκ, μg/ml	6.35 ± 0.12^b^	7.47 ± 0.16^a^
WAP, ng/ml	447.87 ± 14.67	484.14 ± 13.65

CSNαs1, casein alpha s1; CSNαs2, casein alpha s2; CSN2, casein beta; CSN3, casein kappa; WAP, whey acidic protein. a and b denote values that differ significantly at P < 0.05, and A and B denote values that differ significantly at P < 0.01.

### RNA Sequencing and Transcript Analysis

Six cDNA libraries were constructed from porcine mammary tissues exposed to thermal stress or to suitable temperatures. Each RNA-seq library generated approximately 94.52 ± 2.83 million raw reads of 110 nt in length, representing about 3.70 ± 0.11 fold coverage of the porcine genome. After quality control trimming, a total of 88.08 ± 2.65 million valid reads were obtained, accounting for 93.19% ± 0.23% of the raw reads in each library. We aligned all valid reads onto the porcine *Sscrofa11.1* reference genome and found that over 80.55% ± 1.63% of the reads could be mapped successfully to the genome, including 76.13% ± 1.63% of the mapped reads with proper pair alignment in the six libraries (**Table S2A**). In addition, most valid reads were mapped to exons (77.51% ± 2.64%), 17.27% ± 2.02% were mapped to introns, and the rest to intergenic regions (5.22% ± 0.62%), indicating confidence in the quality of library construction and sequencing analysis.

Transcript assemblies from porcine mammary tissue with Cufflinks revealed a total of 133,145 isoforms across six samples, including approximately 37.33% identified candidates that completely matched Ensembl transcript regions (**Table S2B**). A comparison of known Ensembl transcripts revealed that 36,271 isoforms were expressed across all tissues; NS-specific units accounted for 82.08% of known Ensembl isoforms, while 81.37% of the known isoforms existed in the HS libraries (**Table S2C**). Raw Ensembl gene expression levels were quantified by the FPKM algorithm, and the 10 most prevalent functional isoforms accounted for 7.97% ± 0.62% of the total raw reads. In addition, seven gene candidates of *PAEP*, *CSN1S1*, *CSN3*, *CSN2*, *JCHAIN*, *COX1*, and *NUPR1* were shared in the top 10 expressed genes in each sequencing library. These highly expressed isoforms are well-known as having key functions in the lactation process, consistent with the physiological roles of genes expected to be found in mammary gland tissues.

### Identification of circRNA

**Figure 2:**
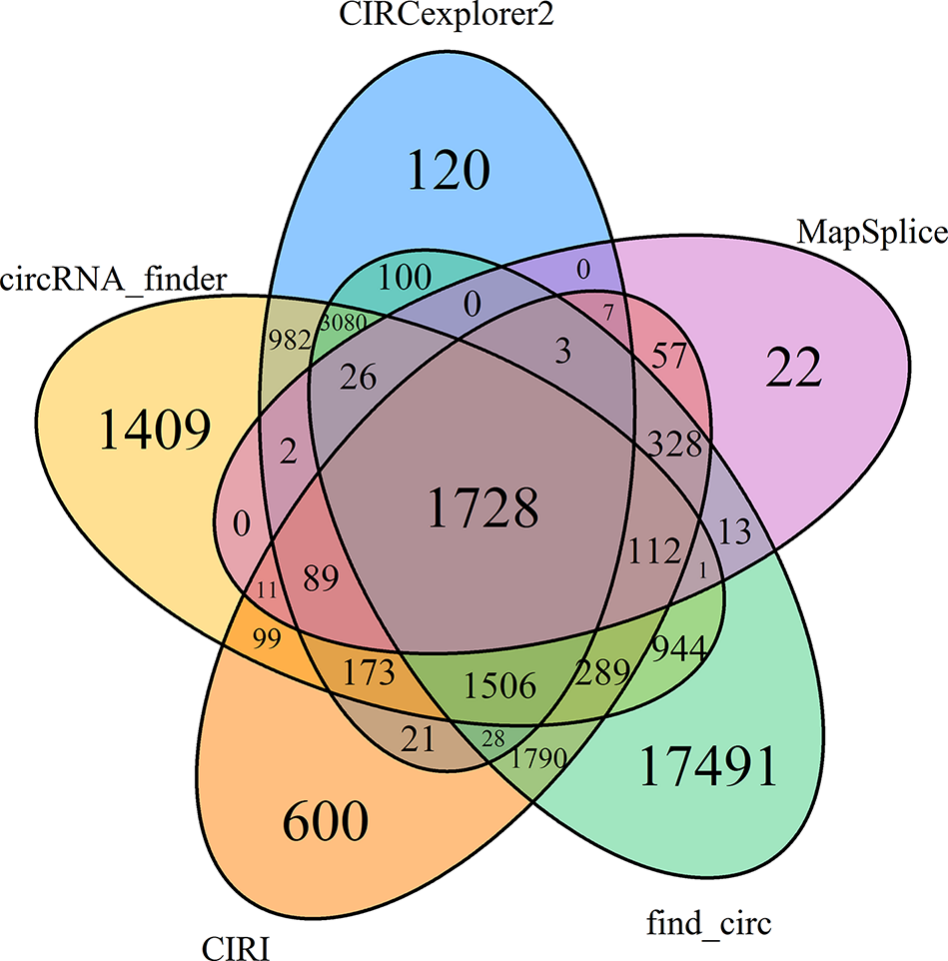
Common circRNA candidates identified by all five approaches.

Several tools have been developed for identification of circRNAs based on back-spliced reads produced from high-throughput RNA sequencing datasets (Hansen et al., 2015). Due to the rearranged exon ordering, these back-spliced events usually cannot be mapped onto the reference genomes (Zhang et al., 2014). In the present study, we identified 17.07 ± 0.25 (19.75% ± 0.80% of the valid reads) and 17.07 ± 3.19 (19.16% ± 3.55%) million unmapped reads in the NS and HS libraries, respectively. Among them, we found a total of 948.00 ± 23.98 thousand back-spliced junction events (1.09% ± 0.04% of the valid reads) in the rRNA-depleted libraries of NS animals, as well as 892.49 ± 80.27 thousand candidates in the HS libraries. We then compared five different circRNA predicting algorithms and found a total of 31,031 unique circRNAs identified across six libraries. Of these, 19,642 were found by a single algorithm, accounting for 63.29% of all the circRNAs identified (**Figure 2**), indicating that the circRNA landscape differs quite dramatically depending on the algorithm used. In particular, find_circ and MapSplice exhibited the highest and lowest level of sensitivity; this is in part reflected in the total number of circRNAs predicted, where find_circ and MapSplice output the highest and lowest number of circRNA species (27,439 and 2,399, respectively) compared to CIRCexplorer2, circRNA_finder, and CIRI methods (7,865, 10,451, and 6,841 species, respectively; **Table S3**). To limit the level of false positive circRNAs, only circRNA candidates identified by all five approaches were considered for further evaluation. Of the 31,031 predicted circRNAs, only a modest overlap of 1,728 circRNAs (5.57%) was observed among the five prediction pipelines. These 1,728 circularization events were found to be produced from 1,157 hosting isoform loci, including 571 transcripts that generated more than one circRNA. For instance, we found that the porcine genes *SEC24A* and *SLC5A10* had nine and eight predicted circRNAs, respectively, and there were seven circularization events predicted from the *BAZ2B*, *PIAS1*, and *CCAR1* genes (**Table S4A**).

### Differential Expression and Functional Analysis

**Figure 3:**
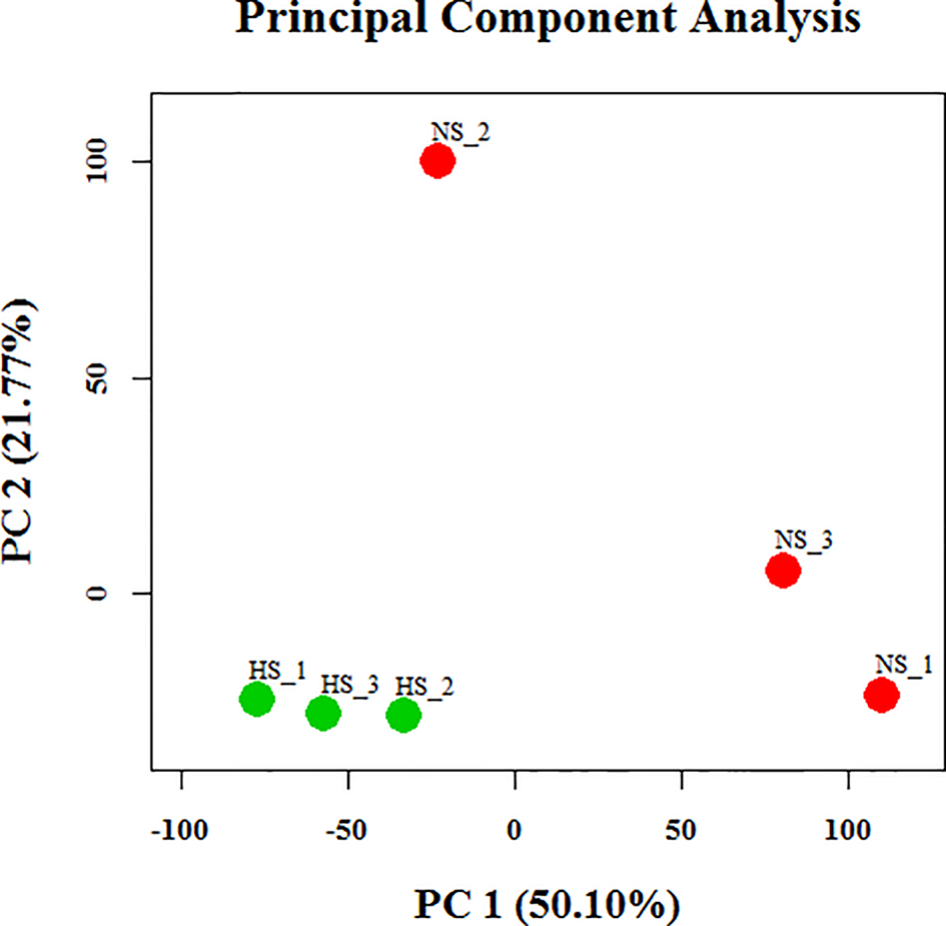
Principal component analysis of assembled transcripts in six libraries. PC, principal component; NS, non-heat stress; HS, heat stress.

To identify dissimilarities between the tested individuals, principal component analysis (PCA) of the globally expressed transcript with the FPKM levels was performed (**Figure 3**). This analysis indicated that the differences in expression between the NS and HS groups were greater than the differences between pools from each particular group. Therefore, we employed the Cuffdiff algorithm to analyze differences in mammary gland gene expression between the NS and HS groups to identify candidate transcripts that are responsive to thermal stress. In our dataset there were over 40,000 unique Ensembl transcripts sequenced, most of which had a very small FPKM value in total across all libraries. In order to filter out false-positive results, we only kept confident transcripts that were expressed in at least three libraries, and finally 9,789 tags were identified in our study (**Table S5A**). Among these, we detected a total of 67 differentially expressed (DE) transcript events by a limited cut-off of FDR < 0.05, representing 63 protein-coding genes, with 42 transcripts increased and 25 transcripts decreased in the HS groups compared to the NS group (**Table S5A**). Analysis of gene ontology (GO) enrichment for DE genes, using identified porcine genes as background in the current experiment, revealed that these genes were significantly enriched in lactation-related functions or stress-inducible biological processes, including “lactation”, “defense response”, “inflammatory response”, “response to stimulus”, and “regulation of immune system process” (**Table S5B**). These 67 DE genes are significantly involved in only one KEGG signaling pathways, termed as toll-like receptor signaling pathway. Although the role of these genes needs to be validated experimentally, the GO and KEGG pathway analyses collectively illustrate some possible avenues to improve HS resistance of lactating sows. In addition, we also clustered differential porcine circRNA expression counts between NS and HS libraries, as determined by the CIRCexplorer2 algorithm, and normalized with trimmed mean of M-values (TMM) (Robinson et al., 2010). In total, only 50 out of 1,728 identified circRNAs were DE, including 29 up-regulated candidates and 21 down-regulated candidates in the NS samples compared with the HS samples (**Table S4B**).

### Functional Interactions Between circRNA and mRNA

To identify possible correlations between circRNA and mRNA expression, we first used the Pearson test and found a total of 1,423 significant interactions between DE circRNAs and DE genes in our study (**Table S6**). In **Table S6** we identified 464 sponge modulators participating in 100 miRNA-mediated regulatory interactions, including 45 circRNAs and 36 unique mRNAs. In addition, the Pearson analysis also revealed 84 significant interactions between DE circRNAs and four lactation-related coding genes (CSN1S1, CSN1S2, CSN3, WAP) that were annotated by GO analyses, and these interactions included 42 positive significant interactions and 42 negative interactions (**Table S6**). We observed that the highest expressed circRNA, *circCSN1S1_2*, was significantly and positively associated with the expression of the *CSN1S1*, *CSN1S2*, *CSN3*, and *WAP* genes; interestingly, these gene products represent the main components of lactoprotein. Recent reports also showed that diverse RNA species can communicate with and co-regulate each other by competing to bind with shared miRNAs, acting as competing endogenous RNAs (ceRNAs) (Tay et al., 2014). We constructed circRNA-miRNA-mRNA networks by pairing the shared miRNA recognition sequences. In total, we generated a network that contained eight nodes and five connections formed between four circRNAs, three miRNAs, and *CSN1S1* gene, including five circRNA-miRNA interactions and three mRNA-miRNA interactions (**Table S7**). Of these, the circCSN1S1_2-miR-204 (miR-670)-CSN1S1 ceRNA axis was established, corresponding well with the ceRNA hypothesis.

### RT-qPCR Analysis

Based on ceRNA networks involved in mammopoiesis and lactogenesis under HS that we constructed, we identified a total of 10 interaction core genes (**Figure S2**). We validated expression of these core genes by RT-qPCR, including eight lactation-related coding genes, one heat-response gene, and circCSN1S1_2, which had the highest expression levels in our study. Divergent and convergent primers were designed for circRNA candidates according to the method described in previous study (Sun et al., 2017). All tested candidate genes showed consistent expression patterns between RT-qPCR and sequencing analysis, suggesting that our estimation of abundance was accurate. Briefly, the expression of coding genes for lactoprotein were significantly lower in HS sows (*P* < 0.05), except for *CSN3*, which decreased in HS sows, but the change was not significant. Usually, HSP family member proteins have important roles as molecular chaperones that help prevent apoptosis under various stress conditions, including HS (Sakatani et al., 2012). In agreement with the sequencing analysis, HSP90AA1 showed a strong induction in response to HS, and it had high expression levels in the HS group. In addition, expression of circCSN1S1_2 was significantly lower in HS sows, demonstrating the validity of our post-transcriptional ceRNA regulatory model.

## Discussion

In general, HS is caused by a combination of environmental temperature, relative humidity, solar radiation, air movement and precipitation, and the majority of studies on HS in livestock have focused mainly on temperature and relative humidity (Bohmanova et al., 2007). In our experiment, the average temperatures and humidity levels during the HS challenge were 32.7 ± 0.40°C and 76.1 ± 0.38%, respectively; thus, the lactating sows in our test might be under HS (Bergsma and Hermesch, 2012). We observed greater concentrations of LDH and HSP70, and a decrease of COR levels in sows under HS conditions, compared with those in an NS climate; these values are generally considered as indicators of stress in pigs (Yu et al., 2010; Belhadj Slimen et al., 2016). Enhanced levels of serum LDH is a biomarker of liver damage in hyperthermic animals (Ozaki et al., 1995). Cao et al. (2011) reported that chronic HS induces significant increases LDH levels in rat plasma; this report was similar to what we observed in lactating sows. HSPs are molecular chaperones that differ in regards to their biological functions and molecular weights (Feder and Hofmann, 1999). Among the various HSP classes, HSP70 levels are associated with the acquisition of thermotolerance (Bedulina et al., 2013). In farm animals, HS significantly increases serum HSP70 concentrations in beef cattle (Gaughan et al., 2013), dairy cow (Min et al., 2015), buffalo (Manjari et al., 2015), sheep (Romero et al., 2013), goat (Dangi et al., 2015), and broiler chicken (Gu et al., 2012); these reports are in excellent agreement with the experimental results in our study. In addition, Wang et al. (2015) reported that acute heat exposure significantly elevated levels of COR in rats, and porcine serum COR levels rapidly increased when individuals were subjected to 40°C for 5 hours (Yu et al., 2010). In contrast, summer temperature-induced HS dramatically repressed COR concentrations in the present study. This finding may be due to the different effects between short-term acute HS and chronic HS. Generally, short-term heat exposure increases plasma COR levels, while long-term exposure decreases them (Du Preez, 2000). Christison and Johnson (1972), and Wiersma and Stott (1974) noted a similar trend in dairy cattle exposed to hot summer conditions. HS has been suggested to be responsible for inducing oxidative stress and immune responses in livestock animals during the summer (Das et al., 2016). In the present study, serum SOD and IgG levels were higher in the HS group than in the NS group, suggesting that the antioxidative and immune function of sows increases to adapt to the adverse environment. Recently, elevated concentrations of SOD and IgG were also reported to be sensitive to ambient temperature in broiler chickens (Azad et al., 2010), lactating buffaloes, and cows (Lallawmkimi et al., 2013; Yatoo et al., 2014). PRL is vital for lactogenesis (Akersr et al., 1981), and concentrations of plasma PRL decrease in dairy cows during thermal stress (Tao et al., 2011). In agreement with previous studies, our data demonstrated a significant reduction in porcine PRL levels due to elevated ambient temperature.

In our study, no significant effects of HS were observed on the number of live piglets born per litter, nor on litter birth weight; similar findings were reported by Lucy and Safranski (2017), who demonstrated no clear influences of gestational HS on the number of piglets born live per litter. The seasonal influences of our study and that have been reported previously on piglet traits at birth are mainly caused by a delayed response to ambient temperature, i.e. sows were mated and conceived during the cool season and subsequently farrowed in the hot season; it is established that the primary effects of temperature on litter traits in piglets primarily occur during the first 4 weeks of gestation (Tummaruk et al., 2004). The HS sows weaned piglets that were approximately 0.5 kg lighter in our study than the NS sows. This represented an about 8.47% decrease in weaning weight, and is in accordance with the results of Williams et al. (2013). In addition, piglets were more susceptible to heat-induced reductions in piglet weight gain during early lactation, in concordance with a study by Spencer et al. (2003), who reported a 17% decrease in piglet weaning weight when lactation period lasted 14 days. We observed a downward trend of high temperature on total milk solids, which were reduced by approximately 8.77%, including specific losses in milk fat and lactose content by about 14.69% and 10.45%, respectively. Generally, milk composition varies considerably throughout the seasons, as showed in multiple farm animals included Holstein cows (Shwartz et al., 2009; Bernabucci et al., 2015), dairy goats (Chornobai et al., 1999), and mares (Markiewicz-Kęszycka et al., 2015). In dairy cows, lower milk fat (Bernabucci et al., 2015) and lactose (Shwartz et al., 2009) values are recorded during the summer months, in correspondence with an increase in Temperature Humidity Index. A similar trend of variation in milk composition was also reported for goat milk, and high air temperature in the summer was significantly negatively correlated with goat milk physico-chemical characteristics (Chornobai et al., 1999). In particular, the milk characteristics most highly affected by air temperature were fat and lactose contents, with correlation coefficients of −0.90 and −0.77, respectively. In contrast, mare milk collected in summer had a significantly higher fat content than in autumn, but the average concentration of lactose was similar for milk collected in summer and in autumn, and showed no specific significant seasonal variations (Markiewicz-Kęszycka et al., 2015). These large discrepancies observed in lactating mares may be due to differences in experimental animals (sows and cows vs. mares) or differences in climate conditions (32.7 ± 0.40°C vs. 23.15 ± 1.61°C in the summer). In agreement with a study comparing lactating sows exposed to high ambient temperature (Renaudeau and Noblet, 2001), our results showed no significant effects of elevated ambient temperature on milk protein, but HS during summer significantly decreased CSNαs1, CSNαs2, and CSNκ concentrations in milk. These results confirmed those obtained by Bernabucci et al. (2015) carried out in dairy cows, in which it was reported that the concentration of CSNα during summer months was 22.6% lower than in the winter, and was 16% lower than in the spring. CSNκ levels were also 9.7% lower during summer than in the winter. Our study agrees with previous studies, and indicates that there is a significant seasonal effect on CSN fractions in domestic livestock milk.

Generally, heat-stressed lactating sows reduce their feed intake, leading to loss of milk production, which can negatively affect piglet growth and development during lactation (Ribeiro et al., 2018). However, Rhoads et al. (2009) and Wheelock et al. (2010) have recently demonstrated that reduced nutrient intake only accounts for about 35%–50% of the HS-induced decrease in milk synthesis. A large portion of the thermal effects on animal lactation may be a consequence of energy intake-independent changes (Wheelock et al., 2010), resulting from genetic regulation of nutrient partitioning during HS (Collier et al., 2008). In the current study, we therefore used RNA-Seq to find the underlying molecular mechanisms of milk synthesis under HS in lactating sows. Sequencing revealed a total of 19,032 unique Ensembl genes in lactating porcine mammary tissues, while genes encoding caseins, whey proteins, and enzymes involved in lactogenesis pathways showed higher expression than other genes with RPKM values. Similar results were obtained in cows (Wickramasinghe et al., 2012), goats (Shi et al., 2015; Crisà et al., 2016), and humans (Lemay et al., 2013). A total of 16,892 genes were expressed in bovine milk somatic cells during early lactation, as well as 19,094 in peak lactation and 18,070 in late lactation, and *LGB* (β-lactoglobulin), *CSN2* (β-casein), *CSN1S1* (α-S1-casein), *LALBA* (α-lactalbumin), *CSN3* (κ-casein), and *CSN1S2* (casein-α-S2) were identified as having the highest expression in milk, based on RPKM values (Wickramasinghe et al., 2012). Approximately 16,024 ovine NCBI unigenes were found to be expressed in mammary glands (Shi et al., 2015), and *CSN2*, *CSN3*, *CSN1S1*, *CSN1S2*, *LALBA*, and *LGB* were the most abundant in the mammary gland transcriptome (Shi et al., 2015; Crisà et al., 2016). In human mammary cells, Lemay et al. (2013) reported 14,629, 14,529, and 13,745 unique genes expressed in colostral, transitional, and mature stages of lactation, and β-casein and α-lactalbumin transcripts made up 45% of the total mRNA expression during lactation. Of the top genes identified in our study, *CSN1S2* had the highest expression among the *CSN* family, followed by *CSN2*, *CSN1S1*, and *CSN3*, which were in discordance with the composition of caseins identified by ELISA analysis. We found that porcine casein-α-S1 constituted up to 51.94% of the caseins in our study. One possibility for this discrepancy was that the abundant caseins are broken into bio-active peptides, and therefore their concentrations are not accurately reflected in the analysis of major milk component proteins. The expression of bio-active peptides formed by cleavage of caseins are higher toward the beginning of lactation (Silva and Malcata, 2005). Another possible explanation is that even though there was high expression of the genes encoding caseins, the protein synthesis may not be efficient in sows that were in negative energy balance, or that were limited in dietary intake of essential amino acids (Wickramasinghe et al., 2012).

In order to further reveal the mechanism of response of lactating sows to HS, we focused on identification of differently expressed genes in response to high ambient temperature. Functional annotation analysis identified that four of these DE genes have principal roles in lactogenesis, including four down-regulated genes (*CSN1S1*, *CSN1S2*, *CSN3*, and *WAP*) in the heat stressed individuals. The CSN1S1, CSN1S2, CSN3, and WAP proteins are the main components of lactoprotein that is usually reduced in response to HS in dairy animals (Bernabucci et al., 2015), and the gene expression analysis was in accordance with the results of the ELISA assay.

Recently, a class of non-coding RNAs, called circRNAs, has been identified across the animal kingdoms (Memczak et al., 2013). These circRNAs usually act as ceRNAs to regulate other coding genes by sharing specific miRNA binding sites (Salmena et al., 2011). Multiple types of circRNA-mediated ceRNA interactions have been linked to various physiological or pathological states, including members of the miRNA-2284 family that are sponged by circCSN1S1 to regulate bovine casein translation (Zhang et al., 2015). Therefore, in the present study we carried out a circRNAome analysis of porcine mammary tissues between NS and HS groups. We identified 50 candidate circRNAs out of 1,728 identified circRNAs that were DE between the NS and HS groups. Based on the ceRNA hypothesis, 42 positively correlated circRNA-mRNA interactions were constructed between the four lactogenic genes and the DE circRNAs using the Pearson algorithm. Of these interaction pairs, analysis by the miRanda application (Enright et al., 2003) revealed four circRNA-mRNA interactions that were predicted to share the same miRNA regulatory elements. In particular, the circCSN1S1_2 binds competitively with miR-204 to increase expression of its hosting gene, *CSN1S1*. A similar ceRNA network was strongly suggested between circFoxo3 and *Foxo3* mRNA in tumor growth and angiogenesis (Yang et al., 2016). Yang et al. reported that circFoxo3 shared identical sequences with the *Foxo3* gene to bind miR-22, miR-136, miR-138, miR-149, miR-433, miR-762, miR-3614-5p, and miR-3622b-5p. These observations indicated that the expression of circRNAs might be related to the expression of their parental genes. Taken together, several circRNA-miRNA-mRNA axes were shown to be likely involved in porcine lactogenesis under HS, and these findings provide novel perspectives on circRNA-associated ceRNA networks for future research in sow lactation.

## Conclusion

We found that constant elevated ambient temperature and HS has negative consequences on piglet growth and performances due to decreased milk production and characteristics of lactating sows. Thermal stress altered genome-wide profiles of circRNAs dramatically in lactating porcine mammary tissue, and these heat-induced circRNAs might participate in mammopoiesis and lactogenesis by post-transcriptional regulation of ceRNA networks. Our results provide novel rationale to investigate circRNA functions in the lactating sow response to HS, and additional research is necessary to quantify and understand these effects.

## Data Availability Statement

All sequencing raw datasets have been deposited into the National Center for Biotechnology Information (NCBI) Sequence Read Archive (SRA) database (https://www.ncbi.nlm.nih.gov/sra/) with the BioProject accession number PRJNA578241.

## Ethics Statement

The animal study was reviewed and approved by the Institutional Animal Care and Use Committee of South China Agricultural University, China.

## Author Contributions

All authors were involved in project conception and design. JS led the lab assays, analyses of data, and writing of the manuscript. JS and YZ contributed reagents, materials, and analysis tools. All authors gave final approval for publication.

## Funding

This research was financially supported by the National Key Research and Development Program of China (2016YFD0500503), the Natural Science Foundation of China Program (31802032), the major scientific projects in general colleges and Universities of Guangdong Province (2017KTSCX023), and the Natural Science Foundation of Guangdong Province (2018B030311015).

## Conflict of Interest

The authors declare that the research was conducted in the absence of any commercial or financial relationships that could be construed as a potential conflict of interest.
