# Supplementary material for: Emerging Roles of Heat-Induced circRNAs Related to Lactogenesis in Lactating Sows
Source: Front Genet. 2020 Feb 11;10:1347. doi: 10.3389/fgene.2019.01347 (PMC7027193; doi:10.3389/fgene.2019.01347)
Supplement: Supplementary file 1 [file Image_1.pdf]

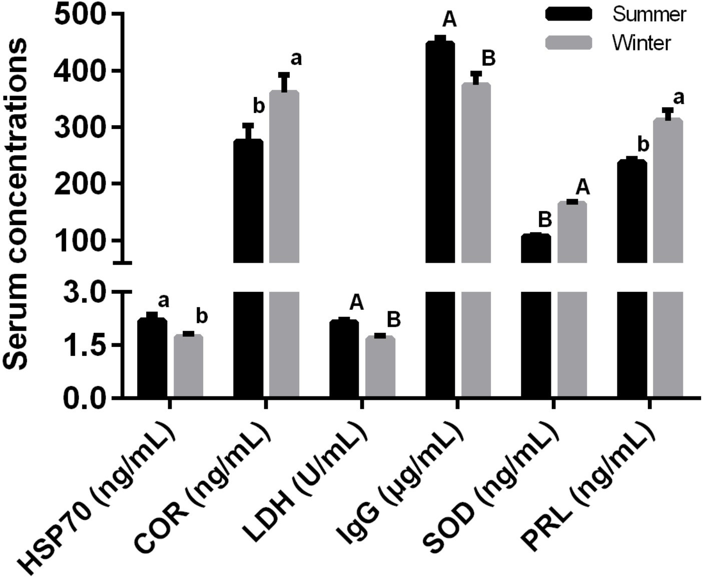

Summer
Winter
Serum concentrations
500
400
300
200
100
3.0
1.5
0.0
a
b
A
B
A
B
B
A
a
b
a
b
HSP70 (ng/mL)
COR (ng/mL)
LDH (U/mL)
IgG (µg/mL)
SOD (ng/mL)
PRL (ng/mL)

Figure S1 Effects of ambient temperature on the serum stress-associated variables in lactating sows

Note: HSP70, Heat shock protein 70; COR, Cortisol; LDH, Lactate dehydrogenase; IgG, Immunoglobulin G; SOD, Superoxide dismutase; PRL, Prolactin; a and b denote values that differ significantly at $P < 0.05$, and A and B denote values that differ significantly at $P < 0.01$.
