# Supplementary material for: Emerging Roles of Heat-Induced circRNAs Related to Lactogenesis in Lactating Sows
Source: Front Genet. 2020 Feb 11;10:1347. doi: 10.3389/fgene.2019.01347 (PMC7027193; doi:10.3389/fgene.2019.01347)
Supplement: Supplementary file 2 [file Image_2.pdf]

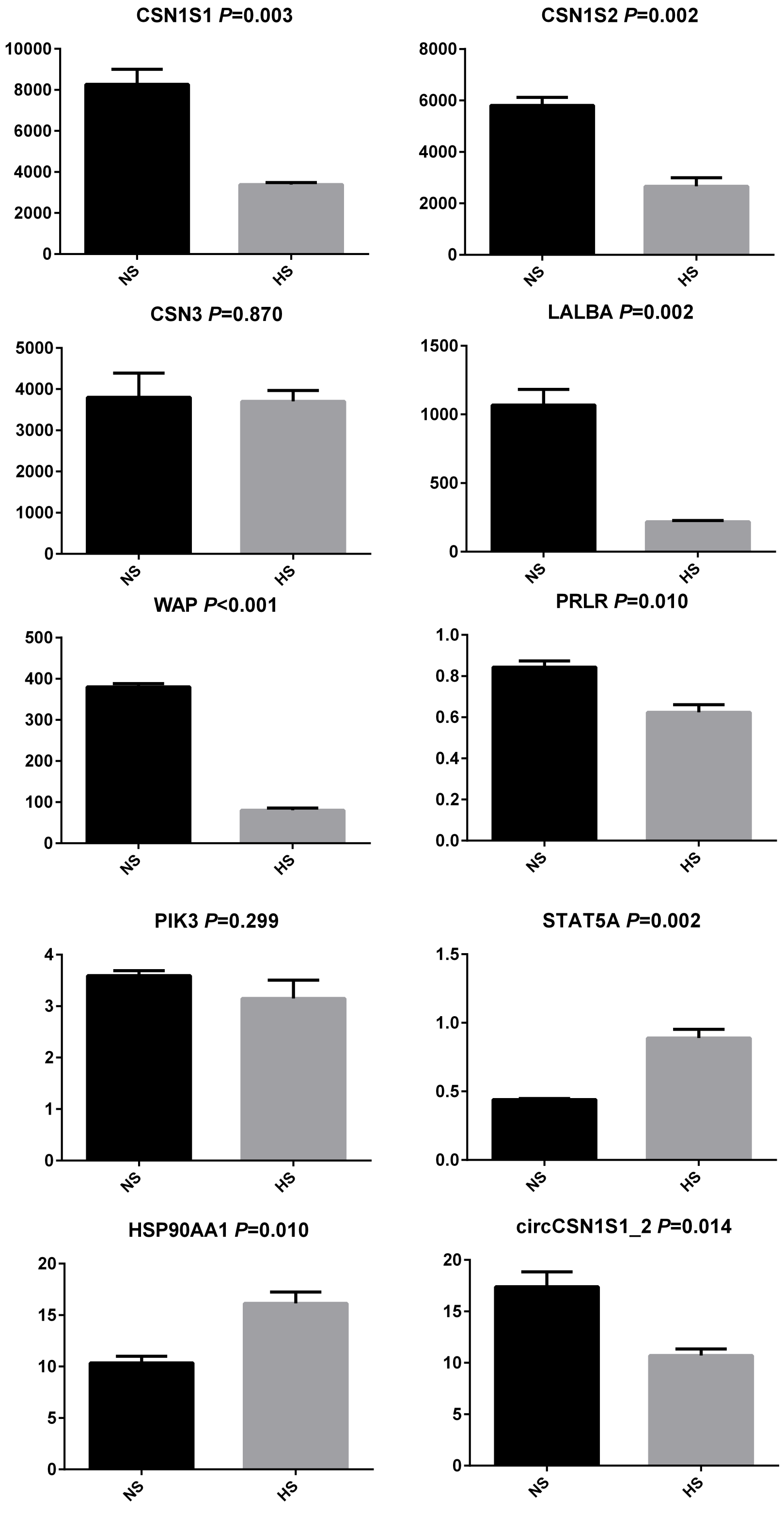
CSN1S1 P=0.003
10000
8000
6000
4000
2000
0
NS
HS
CSN1S2 P=0.002
8000
6000
4000
2000
0
NS
HS
CSN3 P=0.870
5000
4000
3000
2000
1000
0
NS
HS
LALBA P=0.002
1500
1000
500
0
NS
HS
WAP P<0.001
500
400
300
200
100
0
NS
HS
PRLR P=0.010
1.0
0.8
0.6
0.4
0.2
0.0
NS
HS
PIK3 P=0.299
4
3
2
1
0
NS
HS
STAT5A P=0.002
1.5
1.0
0.5
0.0
NS
HS
HSP90AA1 P=0.010
20
15
10
5
0
NS
HS
circCSN1S1_2 P=0.014
20
15
10
5
0
NS
HS

Figure S2 The expression of 8 lactation-related coding genes, one heat-response gene, and circCSN1S1_2

Note: vertical coordinate represented for the relative expression level, and the data represented the mean ± SE from 3 biological replicates with each measurement repeated 3 times.
