# Supplementary material for: Emerging Roles of Heat-Induced circRNAs Related to Lactogenesis in Lactating Sows
Source: Front Genet. 2020 Feb 11;10:1347. doi: 10.3389/fgene.2019.01347 (PMC7027193; doi:10.3389/fgene.2019.01347)
Supplement: Supplementary file 8 [file Table_6.pdf]

Table S6 circRNA→miRNA→mRNA networks identified by the miRanda application

<table>
<tr><th>NO.</th><th>circRNA→miRNA→mRNA network</th><th>circRNA→miRNA interactions</th><th>miRNA→mRNA interactions</th></tr>
<tr><td>1</td><td>circEMSY_1→miR-193a-5p→CSN1S1</td><td>miRNA: 3’ agTAGAGCGGGCGTTTCTGGGt 5’
               ||| | |: | |||||||
circRNA: 5’ ttATCACTGCTAC-AAGACCCa 3’</td><td>miRNA: 3’ agTAGAGCGGGCGT--TTCTGGgt 5’
             ||| :||| |:| ||||||
mRNA: 5’ gtATCATGCCAGTAGGAAGACCac 3’</td></tr>
<tr><td>2</td><td>circCCDC30_1→miR-193a-5p→CSN1S1</td><td>miRNA: 3’ agtagaGCGGGC-GTTTCTGGGt 5’
                  :|| || ::|||||||
circRNA: 5’ gcgagaTGCACGATGAAGACCCa 3’</td><td>miRNA: 3’ agTAGAGCGGGCGT--TTCTGGgt 5’
             ||| :||| |:| ||||||
mRNA: 5’ gtATCATGCCAGTAGGAAGACCac 3’</td></tr>
<tr><td>3</td><td>circCSN1S1_2→miR-204→CSN1S1</td><td>miRNA: 3’ tcCGTA---TCC--TACT-GTTTCCCTt 5’
               |||| ||| |||| |||||||:
circRNA: 5’ tgGCATGGAAGGCCATGAGCAAAGGGGa 3’</td><td>miRNA: 3’ tccGTATCCTACTGTTTCCCTt 5’
              || | || ||:|||||
mRNA: 5’ gacCACTGAAT--CAGAGGGAa 3’</td></tr>
<tr><td>4</td><td>circEPCAM→miR-670→CSN1S1</td><td>miRNA: 3’ gtgaGGACTTACTTATACTCCTTt 5’
                 |||||: | | |||:||||
circRNA: 5’ tggaCCTGAGAGTAAATGGGGAAc 3’</td><td>miRNA: 3’ gtgagGACTTACTT-ATACTCCTTt 5’
               :| |:|||| | | |||||
mRNA: 5’ aagaaTTAAGTGAATTCTCAGGAAc 3’</td></tr>
<tr><td>5</td><td>circCSN1S1_2→miR-670→CSN1S1</td><td>miRNA: 3’ gtgaggactTACTTATACTCCTTt 5’
                      || || :|||||||
circRNA: 5’ tagcagttcATCAA-GTGAGGAAg 3’</td><td>miRNA: 3’ gtgagGACTTACTT-ATACTCCTTt 5’
               :| |:|||| | | |||||
mRNA: 5’ aagaaTTAAGTGAATTCTCAGGAAc 3’</td></tr>
</table>
